# Right ventricular systolic dysfunction in patients with severe ischemic cardiomyopathy - CMR insights into an interventricular relationship

**DOI:** 10.1186/1532-429X-15-S1-P203

**Published:** 2013-01-30

**Authors:** Joao L Cavalcante, Zoran B Popovic, Rory Hachamovitch, Milind Y Desai, Scott D Flamm, Thomas Marwick, Deborah Kwon

**Affiliations:** 1 Heart and Vascular Institute, University of Pittsburgh, Pittsburgh, P, USA; 2 Heart and Vascular Institute, Cleveland Clinic Foundation, Cleveland, OH, USA; 3 Imaging Institute, Cleveland Clinic Foundation, Cleveland, OH, USA; 4 Menzies Research Institute, University of Tasmania, Hobart, TAS, Australia

## Background

Right ventricular systolic dysfunction is associated with worsened outcomes and poor survival in patients with heart failure. However, it is unclear what mechanisms, other than the presence of RV infarction, contribute to the development of RV dysfunction in patients with severe ischemic cardiomyopathy. We sought to determine the impact of baseline demographic variables, CAD severity, LV diastolic function assessed by echocardiography, ventriculovascular coupling, LV remodeling, aortic biomechanical properties, and RV infarction, assessed by CMR, on RV ejection fraction.

## Methods

Patients were selected if they had undergone TTE and CMR studies within 7 days (median=1 day). 354 patients with LVEF ≤ 40% and ≥ 70% stenosis in ≥1 coronary artery but without prior mitral valve surgery, fused E/A waves, atrial fibrillation or > moderate mitral regurgitation were included. Of those, 30 patients were excluded due to suboptimal CMR image quality for adequate RV volume tracings. A total 324 charts were reviewed for demographic and laboratorial data. Diastolic function assessment was performed as per guidelines. Aortic biomechanics were measured using previously validated software (ARTFUN, INSERM U678, Paris, France) using semi-automated tracing of aortic contours with phase-contrast images and through-plane velocity encoding of the ascending and descending aorta. CMR evaluation also included long and short axis assessment of LV/RV function respectively on balanced steady state free precession images along with assessment of LV/RV myocardial scar (on phase-sensitive inversion recovery DHE-CMR sequence ~ 10-20 minutes). Multivariate linear regression analysis performed to identify the independent predictors of RVEF.

## Results

Males represented 73% of the cohort with a mean age of 63 ± 11 years. Mean LVEF was 23 ± 9% and mean RVEF 42 ± 14%. DDFx was classified as either: stage 1 (44%), stage 2 (25%) or stage 3 (31%). The independent predictors of RVEF are listed on Table 1.

**Table 1:** Multivariate predictors of right ventricular ejection fraction

	Unstandardized coefficients		Standardized coefficients		95.0 % Confidence Interval for B	
**Linear Regression Model**	**B**	**Std error**	**Beta**	**P value**	**Lower bound**	**Upper bound**

VVC	14.8	2.192	.331	<0.0001	10.495	19.119
LV Diastolic Dysfunction	-3.879	.586	-.323	<0.0001	-5.031	-2.726
RV Infact by CMR	-3.843	1.212	-.146	0.002	-6.228	-1.458
Gender male	3.902	1.461	.123	.008	1.026	6.777

(*) After adjusting for age, body surface area, glomerular filtration rate, hypertension, diabetes, dyslipidemia, QRS duration, ascending aorta distensibility, LV sphericity, total scar burden, coronary artery disease severity, left ventricular end-systolic volume index. VVC = ventricular-vascular coupling.

## Conclusions

In patients with severe ICM, impaired ventriculovascular coupling and LV diastolic function are associated with RV dysfunction, independent of the presence of RV infarction.

## Funding

None.

